# Urolithin A supplementation alleviates osteogenic disfunction and promotes bone fracture healing in inflammatory environments

**DOI:** 10.29219/fnr.v70.13033

**Published:** 2026-05-21

**Authors:** Jinwu Bai, Jixing Fan, Ruideng Wang, Shilong Su, Daole Hu, Xi He, Shan Gao, Fang Zhou

**Affiliations:** 1 Department of Orthopedics, Peking University Third Hospital, Beijing, China; 2 Beijing Key Laboratory of Advanced Bioadaptable Orthopedic Implants, Peking University Third Hospital, Beijing, China; 3 Department of Trauma Orthopedics, Shenzhen Second People’s Hospital (The First Affiliated Hospital of Shenzhen University), Shenzhen, China; 4 Key Laboratory for Biomechanics and Mechanobiology (Beihang University) of Ministry of Education, Beijing Advanced Innovation Center for Biomedical Engineering, School of Biological Science and Medical Engineering, Beihang University, Beijing, China

**Keywords:** Urolithin A, NF-κB pathway, macrophage polarisation, inflammation, osteogenesis

## Abstract

Excessive and chronic inflammation can cause osteogenic dysfunction and disrupt the balance of the osteoimmune microenvironment, thereby increasing the risk of fracture non-union. Urolithin A (UA), a gut microbiota-derived metabolite produced from dietary sources, has been reported to inhibit RANKL-induced osteoclastogenesis and alleviate postmenopausal osteoporosis. However, the effect of UA on osteogenesis, particularly under pathogenic inflammatory conditions, remains unclear. In this study, mouse bone marrow-derived mesenchymal stromal cells (mBMSCs) were used to evaluate osteogenesis *in vitro*, and RAW 264.7 cells were used as macrophages *in vitro*. Tumour necrosis factor (TNF)-*α* was used to establish an inflammatory environment. *In vivo*, a mouse femur fracture model with local TNF-*α* injection was established, and UA or vehicle was administered by intragastric gavage. The UA showed no obvious effect on cell viability at concentrations ranging from 0 to 10 μM and had no direct effect on the osteogenic differentiation of mBMSCs. TNF-*α* treatment significantly decreased the expression of osteogenesis-related genes and proteins and inhibited calcium deposition, whereas UA reversed this inhibitory effect in a dose-dependent manner. Mechanistically, UA inhibited activation of the TNF-*α*-induced nuclear factor-*κ*B signalling pathway. Furthermore, UA reduced pro-inflammatory cytokine levels and inhibited type-1 macrophage polarisation under TNF-*α*-induced inflammatory conditions. Conditioned medium derived from RAW 264.7 cells stimulated with TNF-*α* after UA treatment promoted the osteogenic differentiation of mBMSCs. *In vivo*, local administration of TNF-*α* significantly impaired bone fracture healing in the mouse femur fracture model, whereas intragastric supplementation with UA improved fracture healing and reduced pro-inflammatory responses. Collectively, these findings demonstrated that UA alleviates osteogenic dysfunction through inhibition of the nuclear factor-*κ*B signalling pathway and regulation of macrophage-mediated inflammation under TNF-*α*–induced inflammatory conditions, thereby promoting osteogenesis and fracture healing.

## Popular scientific summary

- Urolithin A (UA) had not direct effect on the osteogenic differentiation of BMSCs.
- UA alleviated osteogenic disfunction through NF-*κ*B pathway and inhibited inflammation and M1 macrophage polarization in TNF-*α* induced inflammatory environments to promote osteogenesis.
- UA improved impaired bone fracture healing caused by chronic inflammation in vivo.

Bone delayed union and non-union are serious complications of fractures and represent challenging problems in clinical orthopaedics. The incidence of non-union ranges from 1.9 to 4.9%, although this varies considerably across different countries and regions (1, 2). High-energy injury, patient age, infection, diabetes mellitus, obesity, non-steroidal anti-inflammatory drug use, and smoking have been investigated as major aetiological factors contributing to non-union (3). Fracture healing is a complex and continuous process that includes haematoma formation and inflammatory responses, and involves both intracellular and extracellular signalling pathways (4). The microenvironment of the fracture haematoma plays a crucial role in regulating bone healing. An immune response occurs within the fracture haematoma, where bone mesenchymal stromal cells (BMSCs) and macrophages constitute the two most important cell types involved during fracture healing (4). Early and low-level inflammatory responses are beneficial and necessary for promoting fracture healing (5). However, excessive and persistent inflammatory conditions caused by bacterial infections or systemic diseases, such as diabetes and osteoporosis, can inhibit bone formation and easily lead to fracture non-union (6, 7). Therefore, regulating the balance of the immune microenvironment and promoting osteogenesis within the fracture haematoma under inflammatory conditions represent important therapeutic strategies and research directions for enhancing fracture healing and preventing fracture non-union.

**Fig. 1:**
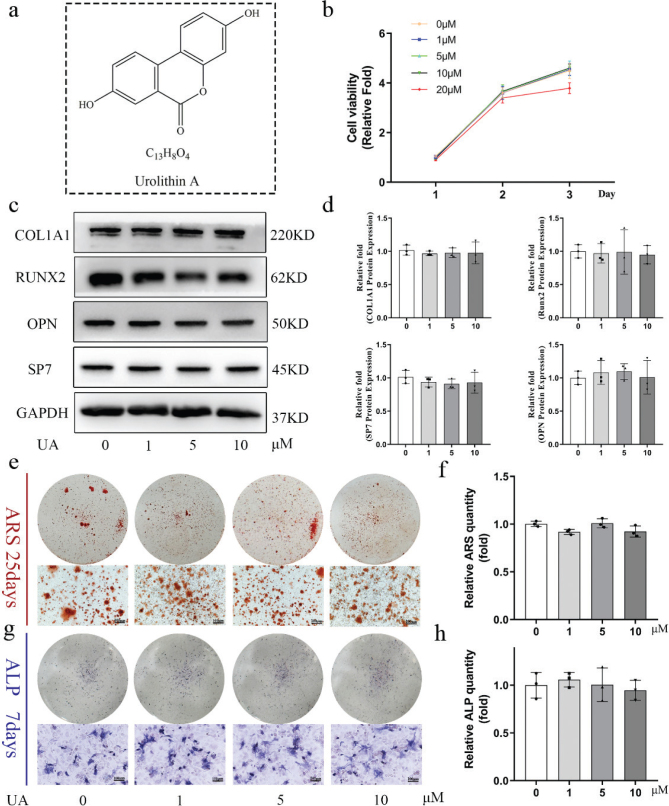
UA has not direct effect on osteogenic differentiation of BMSCs. (a) The chemical structure of UA. (b) CCK-8 assay was utilized to assess the cell viability of BMSCs of UA on 1, 2 and 3 days. (c) The osteogenic related proteins were measured by western blot after 5 days OIM. (d) The quantitative analysis of western blot assay. (e) ARS staining of BMSCs after 25 days OIM. Scale bar, 100 μm. (f) The statistical results of quantitative ARS staining. (g) ALP staining of BMSCs after 7 days OIM. Scale bar, 100 μm. (h) The statistical results of quantitative ALP activity. All of the experiments were independently accomplished by three times. * *P* < 0.05, ** *P* < 0.01 compared to the control group.

Urolithin A (UA) is a natural gut microbiome-derived metabolite produced from ingested ellagitannins and ellagic acid, which are present in dietary products, such as pomegranates, strawberries, raspberries, and walnuts (8, 9). UA belongs to the family of urolithins and is characterised by a chemical structure containing an *α*-benzo-coumarin scaffold (9) (Fig. 1a). Recent research has suggested that UA administration exerts significant beneficial effects in various diseases, including ageing (10), cardiovascular diseases (11), Alzheimer’s disease (12, 13), inflammatory bowel disease (14), diabetes mellitus (15), and non-alcoholic fatty liver disease (16), among others. Furthermore, several preclinical human studies have shown that UA significantly increases muscle strength and provides protection against age-related conditions (17, 18). These effects are mainly attributed to the physiological actions of UA, which enhance cellular health by increasing mitophagy and mitochondrial function while reducing detrimental inflammation (17, 19).

Within the musculoskeletal system, UA has demonstrated protective effects in postmenopausal or age-related osteoporosis (20, 21), osteoarthritis (22, 23), and intervertebral disc degeneration (24, 25). UA has also been shown to significantly inhibit receptor activator of nuclear factor-*κ*B ligand (RANKL)-induced osteoclastogenesis and attenuate systemic inflammation (26–28). However, bone mass regulation depends on the dynamic balance between osteoblast (OB)-driven bone formation and osteoclast (OC)-driven bone resorption (6, 29). The effects and mechanisms of UA on osteogenesis, particularly in inflammatory bone healing models, have not yet been fully explored. Furthermore, the potential role of UA in modulating the interaction between osteogenesis and macrophage polarisation remains unknown.

## Materials and methods

### Reagents and antibodies

Urolithin A (UA, #SJ-MN0067) was obtained from Sparkjade (Shandong, China) and dissolved in dimethyl sulfoxide (MedChemExpress) at a concentration of 20 mM. TNF-*α* (MedChemExpress) was used to establish an inflammatory environment *in vitro*. Antibodies against GAPDH (#2118), RUNX2 (#12556), p-P65 (#3033), P65 (#8242), p-I*κ*B*α* (#2859), I*κ*B*α* (#4812), p-JNK (#4668), JNK (#9252), p-ERK (#4370), ERK (#4695), p-P38 (#4511), and P38 (#9212) were purchased from Cell Signalling Technology (MA, USA). The antibody against iNOS (#bs-0162R) was obtained from Bioss (USA). Antibodies against OPN (#0806-6), SP7 (#HA722817), and COL1A1 (#HA722517) were obtained from HUABIO (Hangzhou, China). The antibody against IL-1*β* (#HY-P80720) was purchased from MedChemExpress.

### Cell extraction, culture, and differentiation

Mouse-derived BMSCs (mBMSCs; #MUBMX-01001) were purchased from Cyagen Biosciences Inc. (Guangzhou, China) and had been previously authenticated. RAW 264.7 cells were obtained from Procell Biotechnology (Wuhan, China). mBMSCs were cultured in *α*-MEM medium (Procell) supplemented with 10% foetal bovine serum (FBS, SA101.02; Cellmax, Beijing, China), while RAW 264.7 cells were cultured in Dulbecco’s modified eagle medium (DMEM)/high-glucose medium supplemented with 10% FBS. For osteogenic differentiation, mBMSCs were cultured in osteogenic induction medium (OIM) as previously described (30), which consisted of low-glucose DMEM supplemented with 10% FBS and three additives: dexamethasone (100 nM), ascorbic acid (0.2 mM), and *β*-glycerophosphate (10 mM) (MedChemExpress). mBMSCs were seeded in 6- or 12-well plates and cultured in OIM with or without UA and TNF-*α* (10 ng/mL). The OIM was replaced every 2 days.

### Cell viability assay

A CCK-8 assay was performed to evaluate cell proliferation. Briefly, cells were seeded in 96-well culture plates and incubated with UA for 1, 2, and 3 days. Subsequently, 10 μL of CCK-8 reagent (Beijing Boxbio Science & Technology Co., Ltd.) was added to each well and incubated for 2 h. The absorbance was measured at 450 nm using a microplate reader.

### Alkaline phosphatase staining and activity assay

After 7 days of osteogenic differentiation, alkaline phosphatase (ALP) staining was performed using an ALP staining kit (Beyotime Biotechnology) according to the manufacturer’s instructions. For ALP activity measurement, activity was assessed using an ALP assay kit (Beyotime), and the total protein content in each well was determined using a BCA protein assay kit (Beyotime).

### Alizarin red staining and quantification assay

After 25 days of osteogenic differentiation, mBMSCs were fixed with 4% paraformaldehyde (PFA) for 15 min. The cells were then washed three times with PBS and ddH_2_O and stained with Alizarin Red solution (0.5%, pH 4.1–4.2; Cyagen Biosciences Inc.) for 20 min. To quantify staining intensity, the stained mineralised nodules were incubated with 10% cetylpyridinium chloride (#SJ-MA0234, Sparkjade). The resulting solution was collected, and absorbance at 570 nm (A570) was measured using a microplate reader.

### Quantitative real-time polymerase chain reaction

Total RNA was extracted from cultured cells using RNAex Pro RNA reagent according to the manufacturer’s protocol. First-strand cDNA was synthesised using the SPARKscript II RT Plus Kit (with gDNA Erase), and real-time polymerase chain reaction (PCR) analyses were performed using 2× SYBR Green qPCR Mix. The mRNA expression levels of the target genes were normalised to those of the housekeeping gene GAPDH. The primer information is presented in Table 1.

**Table 1:** Sequences of primers for real-time quantitative PCR analysis

Gene name	Forward Primer (5′→3′)	Reverse Primer (5′→3′)
Col1a1	GCCGCAAAGAGTCTACATGT	CTTCTTGGCCATGCGTCAG
Runx2	CACCTCGAATGGCAGCACGCTA	GCCGCCAAACAGACTCATCCA
Sp7	CCTAAGGGGCACAGCTCGTCT	TGCATGTCCCACCAAGGAGTAGG
Ocn	CAGTATGGCTTGAAGACCGC	GACATCCATACTTGCAGGGC
Opn	ATCTCACCATTCGGATGAGTCT	TGTAGGGACGATTGGAGTGAAA
Alp	GACCACGGACATCATGAGGGTA	ACCGAATGTGAAAACGTGGGAAT
iNOS	AAACCCCTTGTGCTGTTCTC	GTCTCTGGGTCCTCTGGTCA
CD86	ATGGACCCCAGATGCACCA	CTGTGCCCAAATAGTGCTCG
IL-1β	TGGAGAGTGTGGATCCCAAG	GGTGCTGATGTACCAGTTGG
IL-6	ATAGTCCTTCCTACCCCAATTTCC	GATGAATTGGATGGTCTTGGTCC
GAPDH	GGCAAATTCAACGGCACAGTCAAG	TCGCTCCTGGAAGATGGTGATGG

### Western blot analysis

Cells were lysed on ice using RIPA buffer containing phosphatase and protease inhibitor cocktails (M5293, AbMole, USA). Equal amounts of protein were separated by 10% polyacrylamide gel and transferred onto polyvinylidene fluoride membranes (KeyGEN BioTECH). The membranes were blocked for 1 h with 5% non-fat milk. Subsequently, the membranes were incubated overnight at 4°C with the primary antibodies. Horseradish peroxidase-conjugated anti-rabbit IgG was used as the secondary antibody. Chemiluminescence (Thermo Fisher Scientific, USA) was used to detect the signals, which were quantified by densitometric analysis using ImageJ software.

### Enzyme-linked immunosorbent assay

Inflammatory cytokines in the culture medium of RAW 264.7 cells were measured by enzyme-linked immunosorbent assay (ELISA) using Mouse IL-1*β* (#SEKM-0002) and IL-6 (#SEKM-0007) kits (Solarbio, Beijing, China) according to the manufacturer’s protocol.

### Immunofluorescence staining

After treatment for the indicated time, cells were fixed with PFA for 15 min. The cells were then permeabilised with 0.05% Triton X-100 for 15 min and blocked with 2% bovine serum albumin for 30 min. The fixed cells were washed and incubated overnight with the primary antibody. Subsequently, the cells were incubated with fluorescence-conjugated secondary antibodies (Beijing LABLEAD Inc.) for 1 h, and the nuclei were stained with DAPI (Beijing LABLEAD Inc.) for 10 min. The samples were observed under a fluorescence microscope (Leica, Wetzlar, Germany).

## In vivo evaluation in animals

Animal experiments were approved by the Ethics Committee of Peking University Third Hospital and were performed in accordance with the guidelines of the World Medical Association (WMA) Statement on Animal Use. A mouse femur transverse fracture model was established following our previous study (31). TNF-*α* (100 ng/mL) was used to create an inflammatory environment (5). Briefly, 15 male mice aged 6 weeks were randomly divided into three groups (femur fracture + PBS, femur fracture + TNF-*α*, and femur fracture + TNF-*α* + UA). Firstly, the mice were anaesthetised by inhalation of 2–5% isoflurane, and anaesthesia was maintained with 2% isoflurane inhalation during surgery. A lateral incision was made over the femur, and the muscle was bluntly dissected to expose the femur. The patella was then dislocated, and a sterile 25-gauge needle was inserted into the femoral shaft and subsequently retracted. A transverse fracture in the middle of the femur was created using a micro wire saw. The needle was then positioned through the femur to stabilise the fracture section at the middle and lower segments, created using a custom-made three-point bender. Subsequently, a volume of 20 μL TNF-*α* (100 ng/mL) or normal saline (NS) was injected locally at the fracture site on days 0, 2, 4, and 6. UA (50 mpk) or an equal volume of NS was administered by gavage daily for 1 month after surgery. All mice were sacrificed 1 month after surgery, and the samples were collected and fixed in 4% PFA for 48 h at room temperature.

### Micro-CT and radiographic analysis

The femurs of all mice were scanned and imaged using micro-CT (Bruker SkyScan1176, Billerica, MA) under the following parameters: 70 kVp; reconstruction matrix, 1,024; slice thickness, 14.8 μm; and exposure time, 300 ms. Bone volume analysis was subsequently performed within the region extending 1 mm above and below the fracture ends.

### Histological evaluation and immunohistochemical staining

The fixed femur samples were decalcified and embedded in paraffin. Haematoxylin and eosin (H&E) staining, Masson’s trichrome staining, and immunohistochemistry were performed for micromorphological analysis according to the manufacturers’ protocols. A scoring scale based on cortical debridement and healing acceleration was used for histological quantification (31) (Table 2). All evaluations were conducted in a triple-blind manner.

**Table 2:** Histological evaluation scoring scale of fracture healing

Histological evaluation sites	Scores
No bridging, no woven bone	0
No bridging, a small amount woven bone	1
No bridging, obvious initial woven bone near fracture	2
No bridging, marked woven bone near and around fracture site	3
Bridging of at least one of the cortices, marked woven bone near and around fracture site	4
Bridging of at least one of the cortices, marked and complete woven bone around fracture site	5
Bridging of both cortices, and/or some resolution of the woven bone	6
Clear bridging of both cortices and resolution of the woven bone	7

### Data and statistical analysis

Statistical analysis was performed using Prism (version 8.0; GraphPad Software, San Diego, CA). All experiments were conducted at least three times, and the data were presented as the mean ± standard deviation. Differences between two groups were analysed using the two-tailed Student’s *t*-test. For comparisons involving more than two groups, one-way analysis of variance followed by Bonferroni post hoc tests was used. In all analyses, *P* < 0.05 was considered to indicate statistical significance.

## Results

### UA has no direct effect on the osteogenic differentiation of BMSCs

The chemical structure of UA is shown in Fig. 1a. Firstly, the effect of UA on the viability of mBMSCs was assessed using a CCK-8 assay. No significant effect on cell proliferation was observed at UA concentrations ranging from 0 to 10 μM, whereas a concentration of 20 μM exhibited an obvious inhibitory effect on cell proliferation (Fig. 1b). Therefore, concentrations ranging from 0 to 10 μM were used in the subsequent experiments. We then evaluated the direct effect of UA on osteogenesis. BMSCs were induced with OIM in the presence of different concentrations of UA (0, 1, 5, and 10 μM). After 5 days of OIM induction, the expression of osteogenesis-related genes (Supplementary Fig. 1) and proteins (Fig. 1c, d), including COL1A1, RUNX2, SP7, OPN, ALP, and OCN, showed no significant differences compared to the control group. In addition, Alizarin red staining (ARS) staining (Fig. 1e, f) and ALP staining (Fig. 1g, h) also showed no significant differences following UA treatment. These results indicated that UA has no direct effect on the osteogenic differentiation of BMSCs.

### UA alleviated osteogenic dysfunction of BMSCs caused by TNF-*α*-induced inflammation

**Fig. 2:**
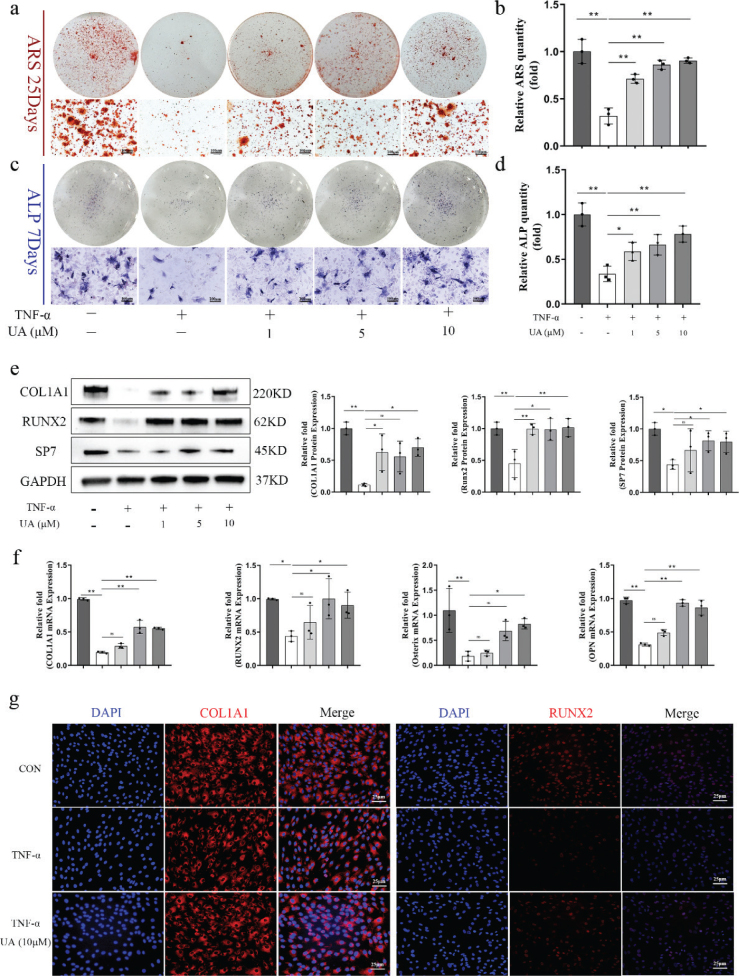
UA alleviated osteogenic dysfunction of BMSCs caused by TNF-*α* induced inflammation. (a, b) ARS staining of BMSCs after 25 days OIM and quantitative result. Scale bar, 100 μm. (c, d) ALP staining of BMSCs after 7 days OIM and ALP activity quantitative result. Scale bar, 100 μm. (e) The osteogenic related proteins were measured by western blot after 7 days OIM and quantitative analysis. (f) The osteogenic related genes were measured by qRT-PCR after 7 days OIM. (g) Immunofluorescence staining for COL1A1 and RUNX2 after 7 days OIM. Scale bars, 25 μm. All of the experiments were independently accomplished by three times. * *P* < 0.05, ** *P* < 0.01 compared to the control group.

A prolonged and excessive inflammatory microenvironment can impair osteogenesis during bone fracture healing. To explore further the mechanism of UA in osteogenesis under an inflammatory environment, TNF-*α* (10 ng/mL) was used to create an inflammatory condition during the osteogenic differentiation of BMSCs. ARS and ALP staining revealed that TNF-*α* treatment significantly inhibited calcium deposition and ALP activity in BMSCs. However, UA reversed this inhibitory effect in a dose-dependent manner (Fig. 2a–d). To evaluate further the expression of osteogenesis-related genes and proteins during osteogenic differentiation under TNF-*α* stimulation, Western blotting, immunofluorescence, and qRT-PCR assays were performed. Western blot analysis revealed that the expression levels of osteogenic-specific proteins, including COL1A1, RUNX2, and SP7, decreased significantly under TNF-*α*-induced inflammatory conditions after 7 days of OIM induction, whereas UA treatment alleviated this decrease in BMSCs (Fig. 2e). Consistently, qRT-PCR results also showed that the expression of osteogenesis-related genes increased following UA treatment under TNF-*α*-induced inflammatory conditions (Fig. 2f). Furthermore, immunofluorescence analysis of COL1A1 and RUNX2 expression showed similar results (Fig. 2g). These findings indicated that UA can alleviate osteogenic dysfunction of BMSCs caused by TNF-*α*-induced inflammation.

### UA inhibited TNF-*α*-induced activation of the NF-*κ*B signalling pathway during the osteogenic differentiation of BMSCs

**Fig. 3:**
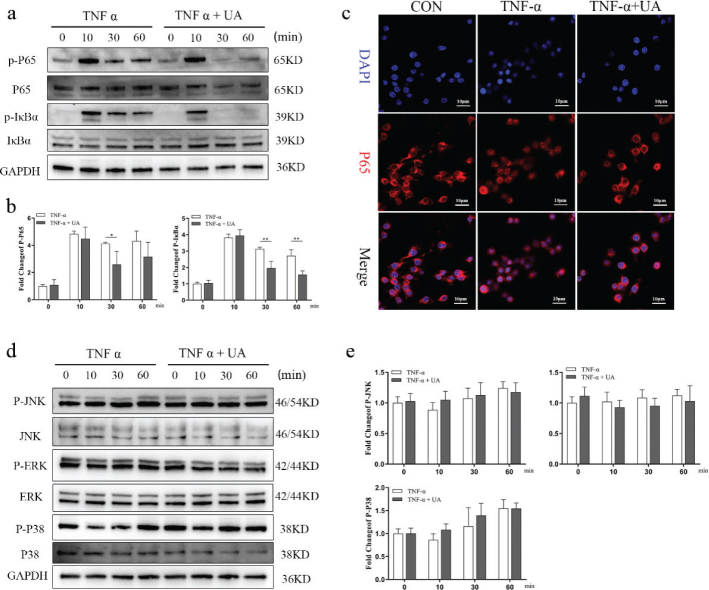
UA inhibited TNF-*α* induced NF-*κ*B signalling pathway activation during the osteogenic differentiation of BMSCs. (a) representative Western blot images of p-p65, p65, p-I*κ*B*α* and I*κ*B*α* after TNF-*α* stimulation with or without UA (10 μM) after 0, 10, 30 and 60 min OIM. (b) Quantification of p-p65 and p-I*κ*B*α* relative to GAPDH. (c) Immunofluorescence staining for p-P65 after 60 min of OIM. Scale bars, 10 μm. (d) Representative Western blot images of p-JNK, JNK, p-ERK, ERK, p-P38 and P38 after TNF-*α* stimulation with or without UA (10 μM) after 0, 10, 30 and 60 min OIM. (e) Quantification of p-JNK, p-ERK and p-P38 relative to GAPDH. All of the experiments were independently accomplished by three times. * *P* < 0.05, ** *P* < 0.01 compared to the control group.

To investigate further the potential mechanism of UA during osteogenic differentiation under TNF-*α*-induced inflammatory conditions, the key proteins of the MAPK and NF-*κ*B signalling pathways associated with inflammatory stimulation were measured by Western blot analysis. BMSCs were cultured in OIM with or without UA under TNF-*α* stimulation for 0, 10, 30, and 60 min. The expression levels of phosphorylated p65 (p-p65) and phosphorylated I*κ*B*α* (p-I*κ*B*α*) were significantly decreased by UA treatment under TNF-*α*-induced inflammatory conditions after 30 min (Fig. 3a, b), whereas the expression levels of total p65 and I*κ*B*α* showed no significant differences. Immunofluorescence results further showed that p-p65 exhibited significant nuclear translocation under TNF-*α* stimulation, whereas UA treatment reduced the nuclear translocation of p-p65 (Fig. 3c). In addition, the expression levels of key proteins in the MAPK signalling pathway, including P38, JNK, and ERK, did not show obvious differences following UA treatment (Fig. 3d, e). These results suggested that UA inhibits TNF-*α*-induced activation of the NF-*κ*B signalling pathway during the osteogenic differentiation of BMSCs.

### UA alleviated pro-inflammatory levels and type-1 macrophage (M1) Activation under a TNF-*α*-induced inflammatory microenvironment

**Fig. 4:**
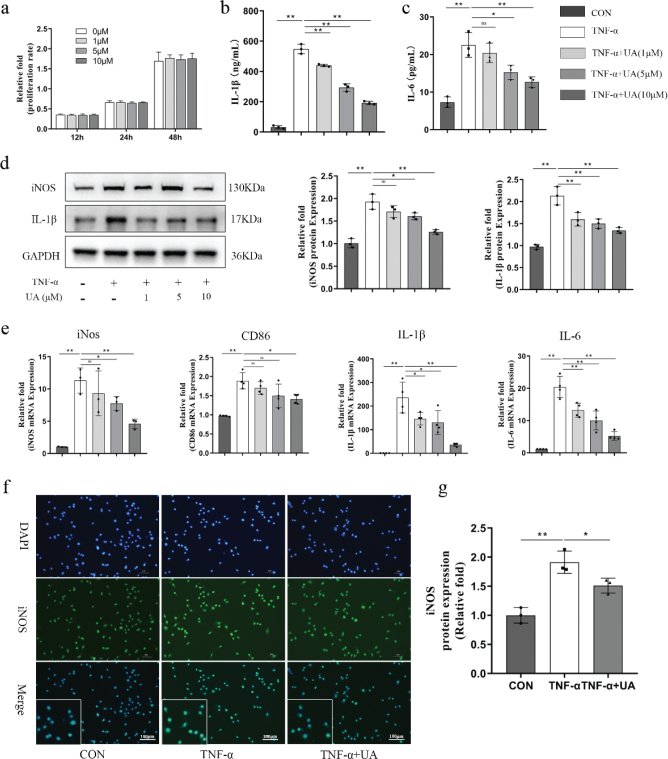
UA alleviated pro-inflammation level and the M1 macrophage activation under TNF-*α* induced inflammation microenvironment. (a) CCK-8 assay was utilised to assess the cell viability of RAW264.7 of UA on 12, 24 and 48 h. (b, c) The concentration of IL-1*β* and IL-6 in the medium of RAW264.7 after 12 h TNF-*α* stimulation with or without UA by ELISA. (d) The proteins of RAW264.7 were measured by western blot after 1 day and quantitative analysis. (e) The genes were measured by qRT-PCR after 1 day. (f) Immunofluorescence staining for iNOS after 1 day after TNF-*α* stimulation with or without UA. Scale bars, 100 μm. (g) The quantitative analysis of Immunofluorescence by image J. All of the experiments were independently accomplished by three times. * *P* < 0.05, ** *P* < 0.01 compared to the control group.

Osteoimmunomodulation mediated by immune cells, particularly macrophages, plays a pivotal role in osseointegration by releasing active factors that improve the inflammatory microenvironment. To evaluate the immunomodulatory capacity of UA under inflammatory conditions, RAW 264.7 cells were used as macrophages *in vitro*. Firstly, a CCK-8 assay was performed to evaluate the effect of UA on cell viability. The results showed that UA at concentrations ranging from 0 to 10 μM had no significant effect on cell proliferation (Fig. 4a). Next, the expression levels of inflammatory cytokines and M1 markers were measured following UA treatment under TNF-*α* stimulation using ELISA, Western blotting, qRT-PCR, and immunofluorescence assays. The levels of the pro-inflammatory cytokines IL-1*β* and IL-6 increased significantly after TNF-*α* stimulation, whereas UA treatment inhibited this effect in a dose-dependent manner (Fig. 4b, c). Similarly, the expression levels of M1 markers, including iNOS and CD86, were markedly increased by TNF-*α* stimulation, and UA partially alleviated this increase (Fig. 4d, e). Immunofluorescence analysis also showed that the increased expression of iNOS induced by TNF-*α* was inhibited by UA treatment, with a significant difference observed (Fig. 4f, g). These results indicated that UA can effectively alleviate the expression of pro-inflammatory cytokines and M1-related proteins induced by TNF-*α* treatment.

### Conditioned medium from RAW 264.7 cells treated with UA under TNF-*α* stimulation promotes osteogenesis of mBMSCs

**Fig. 5:**
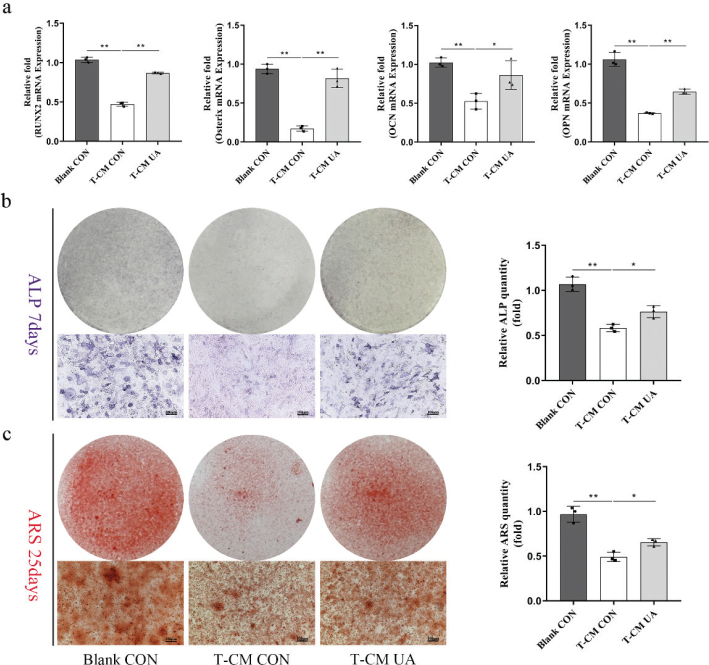
Conditional-medium from RAW 264.7 with UA by TNF-*α* stimulation promote osteogenesis of mBMSCs. (a) The osteogenic related genes measured by qRT-PCR after 7 days. (b) ALP staining of BMSCs after 7 days OIM and ALP activity quantitative result. Scale bar, 100 μm. (c) ARS staining of BMSCs after 25 days OIM and quantitative result. Scale bar, 100 μm. All of the experiments were independently accomplished by three times. * *P* < 0.05, ** *P* < 0.01 compared to the control group.

We previously demonstrated that UA could inhibit M1 activation and downregulate pro-inflammatory responses induced by TNF-*α*. Therefore, we further explored the effect of UA-treated RAW 264.7 cells on the osteogenesis of mBMSCs using an indirect co-culture assay. RAW 264.7 cells were cultured with or without UA under TNF-*α* stimulation. The CM, comprising RAW 264.7 culture medium and OIM at a ratio of 1:1, was prepared and applied to induce osteogenesis in mBMSCs. To eliminate the potential bias caused by different UA concentrations, the final UA concentration in the CM was kept identical among the groups. The osteogenic capability of mBMSCs cultured in CM was assessed by qRT-PCR, ARS staining, and ALP staining. The expression levels of osteogenesis-related genes were significantly downregulated in the TNF-*α*-induced CM without UA, compared to the blank group, whereas TNF-*α*-induced CM with UA partially reversed this effect (Fig. 5a). In addition, ARS and ALP staining showed similar results. CM from TNF-*α*-stimulated RAW 264.7 cells treated with UA significantly increased calcium nodule deposition and ALP activity compared to CM from TNF-*α*-stimulated RAW 264.7 cells without UA (Fig. 5b, c). These findings indicated that CM derived from RAW 264.7 cells treated with UA under TNF-*α* stimulation promotes osteogenic differentiation and calcium deposition. This result further suggested that the osteoimmunomodulatory effect of UA can influence BMSCs and promote osteogenesis under a TNF-*α*-induced inflammatory microenvironment.

### UA accelerated TNF-*α*-induced impaired bone fracture healing in a mouse femur fracture model

**Fig. 6:**
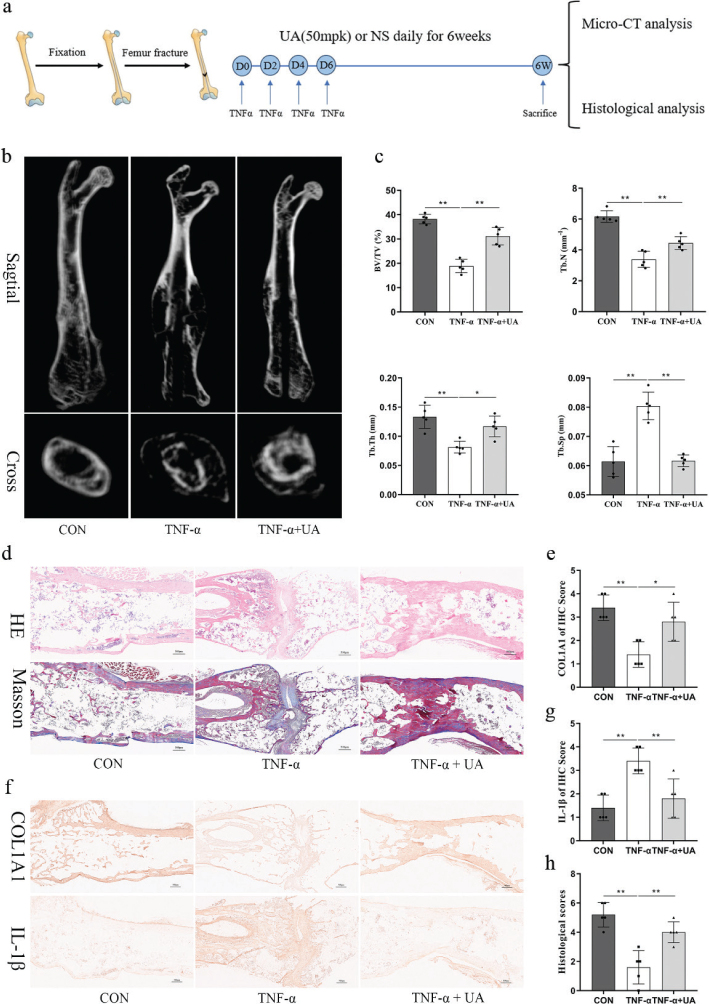
UA accelerated TNF-*α* induced impaired bone fracture healing in a mice femur fracture model. (a) Experiment flow about animal study. (b) The representative μCT images of mouse femurs from the femur fracture + PBS, femur fracture + TNF-*α* and femur fracture + TNF-*α* + UA (50 mpk) group. (c) Graphic illustrations of BV/TV, Tb.N, Tb.Th and Tb.Sp in the indicated groups. (d) Histological section from femur fracture region stained with H&E and Masson staining. Scale bar, 500 μm. (e) Immunohistochemistry of COL1A1 and IL-1*β* of femur fracture region. Scale bar, 500 μm. (f, g) The quantitative results of immunohistochemistry by image J. (h) Histological scores of three groups. All of the experiments were independently accomplished by five times. All error bars represent SDs. * *P* < 0.05, ** *P* < 0.01 compared to the control group.

To investigate further the therapeutic effect of UA on TNF-*α*-induced impaired bone fracture healing, a mouse femur fracture model was established (Fig. 6a). TNF-*α* (100 ng/mL) was locally administered on the day of surgery and injected repeatedly on days 2, 4, and 6 after surgery to create an excessive inflammatory environment. UA (50 mpk) or an equal volume of NS was administered by gavage daily for 1 month after surgery. After 1-month, micro-CT was used to analyse bone mass in the fracture region. The results showed that local administration of TNF-*α* (100 ng/mL) significantly impaired bone fracture healing (Fig. 6b), as evidenced by lower bone volume/total volume, trabecular number, and trabecular thickness and higher trabecular separation. In contrast, UA intragastric administration promoted fracture healing and improved bone mass in the fracture region (Fig. 6c). H&E and Masson staining were performed for histological analysis. UA treatment significantly promoted bridging of both cortices and increased the formation of woven bone near and around the fracture site compared to the TNF-*α* group (Fig. 6d). Consistently, histological evaluation scores were higher in the TNF-*α* + UA group than in the TNF-*α* group (Fig. 6h). To evaluate further the expression of osteogenesis- and inflammation-related markers, COL1A1 and IL-1*β* were examined by immunohistochemistry. The results showed that local administration of TNF-*α* (100 ng/mL) significantly downregulated COL1A1 expression and upregulated IL-1*β* expression, whereas UA treatment reversed these effects (Fig. 6e–g). These findings demonstrated that persistent TNF-*α*-induced inflammation in the local fracture region impairs bone fracture healing while UA treatment can accelerate the healing process by promoting osteogenesis and inhibiting inflammation in a mouse femur fracture model.

## Discussion

The regulation of BMSC osteogenesis and the osteoimmune function of macrophages are crucial for bone fracture healing. Bone fractures are among the most common injuries of the musculoskeletal system, and they are associated with a relatively high incidence of delayed union or non-union due to infection or pathogenic inflammatory conditions, such as osteoporosis, aging, and diabetes. Excessive and chronic inflammation inhibits the osteogenic differentiation of BMSCs and further promotes macrophage polarisation towards the M1 phenotype, thereby amplifying the inflammatory response and ultimately impairing bone fracture healing. Therefore, restoring osteogenic function and maintaining controlled inflammation have become crucial strategies for the treatment and prevention of bone fracture non-union.

**Fig. 7:**
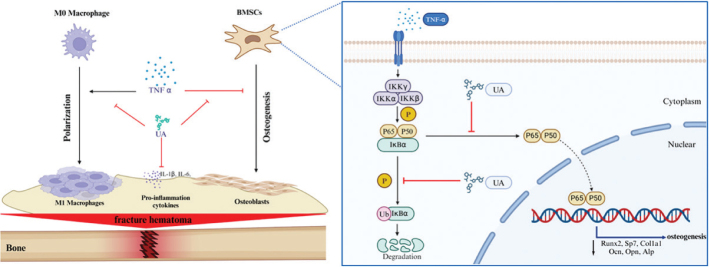
A schematic diagram showing the working model of the role of Urolithin A protect osteogenesis disfunction from excess inflammation.

As a gut metabolite derived from ellagic acid-rich foods, UA has been shown to extend health span and lifespan in animal models (17). Furthermore, several clinical trials have demonstrated that UA can alleviate heart failure (32) and improve muscle strength and endurance (33). UA also exhibits strong anti-inflammatory and antioxidative stress effects. Most studies investigating the role of UA in bone metabolism have mainly focused on OC-mediated bone resorption and osteoporosis. Multiple studies have shown that UA attenuates RANKL-induced osteoclastogenesis and protects against postmenopausal and senile osteoporosis (20, 21, 28). However, there have been no studies examining the effects of UA on OB-mediated bone formation, particularly under chronic inflammatory conditions. Although our study showed that UA has no direct effect on the osteogenic differentiation of BMSCs, UA significantly restored the impaired osteogenic capacity of BMSCs caused by TNF-*α*-induced inflammation. We demonstrated that high-dose TNF-*α* treatment markedly downregulated osteogenesis-related genes and proteins and reduced the mineralisation capacity of BMSCs, whereas UA supplementation restored the impaired osteogenesis. We further explored the potential mechanism and found that UA inhibited activation of the NF-*κ*B signalling pathway during the osteogenic differentiation of BMSCs under TNF-*α*-induced inflammatory conditions, whereas the MAPK signalling pathway showed no significant differences. NF-*κ*B is a transcription factor that regulates the expression of genes involved in cell proliferation and apoptosis, as well as genes associated with inflammatory and immune responses (34). The canonical NF-*κ*B pathway is typically activated through the binding of ligands to their receptors on the cell surface, such as RANK, TNFR, and IL-1R, which subsequently activate the inhibitor of kappa B kinase (IKK) complex composed of the catalytic subunits IKK*α* and IKK*β* and the regulatory subunit IKK*γ*. Upon activation, IKK phosphorylates I*κ*B*α* at two N-terminal serine residues, thereby triggering ubiquitin-dependent degradation of I*κ*B*α* in the proteasome. This process results in rapid and transient nuclear translocation of canonical NF-*κ*B members, predominantly the p50/RelA and p50/c-Rel dimers, which subsequently activate target gene transcription (35). NF-*κ*B activation promotes osteoclastogenesis and inhibits the expression of osteogenesis-specific genes (36). Our study demonstrated that UA inhibited activation of the NF-*κ*B signalling pathway during the osteogenic differentiation of BMSCs under TNF-*α*-induced inflammatory conditions (Fig. 7).

The fracture haematoma, which contains immune cells and multiple signalling factors, plays an important role in bone fracture healing. Early inflammatory events following fracture are critical for the outcome of fracture healing, and inflammation is necessary to initiate the reparative response after injury (7). Low-dose pro-inflammatory cytokines, particularly TNF-*α*, are beneficial and necessary for fracture healing (7). However, excessive and persistent inflammation inhibits osteogenesis and impairs bone healing (37). Moussa et al. (38) reported that UA alleviates systemic inflammation and improves the inflammatory microenvironment in the colon. In our study, UA decreased the levels of pro-inflammatory cytokines in RAW 264.7 cells under TNF-*α*-induced inflammatory conditions *in vitro*. Furthermore, UA administered intragastrically reduced the elevated expression of IL-1*β* in a TNF-*α*-induced femur fracture model *in vivo*. These findings indicated that UA can regulate excessive inflammation during local bone fracture healing. Macrophages are the most important immune cells in the fracture haematoma microenvironment. As key regulators of the immune response, macrophages exhibit remarkable plasticity and can polarise into pro-inflammatory (M1) and anti-inflammatory (M2) phenotypes, each playing distinct roles during osteogenesis (39). TNF-*α* induces macrophage polarisation towards the M1 phenotype, whereas UA was able to reverse this effect. Furthermore, conditioned medium (CM) derived from RAW 264.7 cells treated with UA under TNF-*α* stimulation promoted the osteogenic differentiation of BMSCs. These findings indicated that UA regulates macrophage polarisation and promotes osteogenesis (Fig. 7).

To verify further the therapeutic effect of UA *in vivo*, we established a mouse femur fracture model. An excessive inflammatory condition was induced by local injection of TNF-*α*. Persistent TNF-*α*-induced inflammation significantly impaired bone fracture healing. However, intragastric administration of UA promoted fracture healing and improved bone mass in the fracture region. These findings suggested that UA may represent an economical and effective oral nutritional intervention strategy for the treatment of fracture non-union.

Nevertheless, this study had several limitations. Firstly, the mechanisms underlying the effects of UA on osteogenesis and macrophage polarisation were not investigated in detail. In this study, the mechanism of UA was mainly explored at the level of upstream signalling pathways. Secondly, additional models of chronic systemic inflammatory fracture repair were not included. Future studies will investigate additional disease models to evaluate further the therapeutic potential of UA in fracture healing.

## Conclusions

UA had no direct effect on the osteogenic differentiation of BMSCs. However, UA alleviated osteogenic dysfunction through modulation of the NF-*κ*B signalling pathway and inhibited inflammation and M1 polarisation under TNF-*α*-induced inflammatory conditions, thereby promoting osteogenesis. In addition, UA accelerated impaired bone fracture healing caused by chronic inflammation *in vivo*. These findings suggest that UA is a promising and cost-effective candidate for the treatment of fracture non-union associated with chronic inflammation.

## Supplementary Material



## Data Availability

No datasets were generated or analysed during this study. The original data used during this study are available from the corresponding author upon reasonable request.

## Abbreviations

mBMSC: mouse bone marrow mesenchymal stomal cell; OIM: osteogenic induced medium; OB: osteoblast; OC: osteoclast; TNF-*α*: tumour necrosis factor-*α*; FBS: foetal bovine serum; NF-*κ*B: nuclear factor-*κ*B; RANKL: receptor activator of nuclear factor-*κ*B ligand; IKK: inhibitor of kappa B kinase; I*κ*B*α*: inhibitor of *κ*B-*α*; PFA: paraformaldehyde.

## References

[1] Mills LA, Aitken SA, Simpson A. The risk of non-union per fracture: current myths and revised figures from a population of over 4 million adults. Acta Orthop 2017; 88(4): 434–9. doi: 10.1080/17453674.2017.1321351 28508682 PMC5499337

[2] Zura R, Xiong Z, Einhorn T, Watson JT, Ostrum RF, Prayson MJ, et al. Epidemiology of fracture nonunion in 18 human bones. JAMA Surg 2016; 151(11): e162775. doi: 10.1001/jamasurg.2016.2775 27603155

[3] Nicholson JA, Makaram N, Simpson A, Keating JF. Fracture nonunion in long bones: a literature review of risk factors and surgical management. Injury 2021; 52 Suppl 2: S3–11. doi: 10.1016/j.injury.2020.11.029 33221036

[4] Bai J, Zhang W, Zhou C, Zhao G, Zhong H, Hang K, et al. MFG-E8 promotes osteogenic differentiation of human bone marrow mesenchymal stem cells through GSK3beta/beta-catenin signaling pathway. FASEB J 2023; 37(6): e22950. doi: 10.1096/fj.202201417RRR 37144883

[5] Hang K, Wang Y, Bai J, Wang Z, Wu W, Zhu W, et al. Chaperone-mediated autophagy protects the bone formation from excessive inflammation through PI3K/AKT/GSK3beta/beta-catenin pathway. FASEB J 2024; 38(10): e23646. doi: 10.1096/fj.202302425R 38795328

[6] Wang Z, Wu J, Li L, Wang K, Wu X, Chen H, et al. Eicosapentaenoic acid supplementation modulates the osteoblast/osteoclast balance in inflammatory environments and protects against estrogen deficiency-induced bone loss in mice. Clin Nutr 2023; 42(9): 1715–27. doi: 10.1016/j.clnu.2023.07.022 37542949

[7] Chan JK, Glass GE, Ersek A, Freidin A, Williams GA, Gowers K, et al. Low-dose TNF augments fracture healing in normal and osteoporotic bone by up-regulating the innate immune response. EMBO Mol Med 2015; 7(5): 547–61. doi: 10.15252/emmm.201404487 25770819 PMC4492816

[8] Toney AM, Fox D, Chaidez V, Ramer-Tait AE, Chung S. Immunomodulatory role of urolithin A on metabolic diseases. Biomedicines 2021; 9(2): 192–210. doi: 10.3390/biomedicines9020192 33671880 PMC7918969

[9] D’Amico D, Andreux PA, Valdes P, Singh A, Rinsch C, Auwerx J. Impact of the natural compound urolithin a on health, disease, and aging. Trends Mol Med 2021; 27(7): 687–99. doi: 10.1016/j.molmed.2021.04.009 34030963

[10] Ryu D, Mouchiroud L, Andreux PA, Katsyuba E, Moullan N, Nicolet-Dit-Felix AA, et al. Urolithin A induces mitophagy and prolongs lifespan in C. elegans and increases muscle function in rodents. Nat Med 2016; 22(8): 879–88. doi: 10.1038/nm.4132 27400265

[11] Cui GH, Chen WQ, Shen ZY. Urolithin A shows anti-atherosclerotic activity via activation of class B scavenger receptor and activation of Nef2 signaling pathway. Pharmacol Rep 2018; 70(3): 519–24. doi: 10.1016/j.pharep.2017.04.020 29660655

[12] Fang EF, Hou Y, Palikaras K, Adriaanse BA, Kerr JS, Yang B, et al. Mitophagy inhibits amyloid-beta and tau pathology and reverses cognitive deficits in models of Alzheimer’s disease. Nat Neurosci 2019; 22(3): 401–12. doi: 10.1038/s41593-018-0332-9 30742114 PMC6693625

[13] Shen PX, Li X, Deng SY, Zhao L, Zhang YY, Deng X, et al. Urolithin A ameliorates experimental autoimmune encephalomyelitis by targeting aryl hydrocarbon receptor. EBioMedicine 2021; 64: 103227. doi: 10.1016/j.ebiom.2021.103227 33530002 PMC7851346

[14] Larrosa M, Gonzalez-Sarrias A, Yanez-Gascon MJ, Selma MV, Azorin-Ortuno M, Toti S, et al. Anti-inflammatory properties of a pomegranate extract and its metabolite urolithin-A in a colitis rat model and the effect of colon inflammation on phenolic metabolism. J Nutr Biochem 2010; 21(8): 717–25. doi: 10.1016/j.jnutbio.2009.04.012 19616930

[15] Tuohetaerbaike B, Zhang Y, Tian Y, Zhang NN, Kang J, Mao X, et al. Pancreas protective effects of Urolithin A on type 2 diabetic mice induced by high fat and streptozotocin via regulating autophagy and AKT/mTOR signaling pathway. J Ethnopharmacol 2020; 250: 112479. doi: 10.1016/j.jep.2019.112479 31846746

[16] Toney AM, Fan R, Xian Y, Chaidez V, Ramer-Tait AE, Chung S. Urolithin A, a gut metabolite, improves insulin sensitivity through augmentation of mitochondrial function and biogenesis. Obesity (Silver Spring) 2019; 27(4): 612–20. doi: 10.1002/oby.22404 30768775

[17] Kuerec AH, Lim XK, Khoo AL, Sandalova E, Guan L, Feng L, et al. Targeting aging with urolithin A in humans: a systematic review. Ageing Res Rev 2024; 100: 102406. doi: 10.1016/j.arr.2024.102406 39002645

[18] Singh A, D’Amico D, Andreux PA, Fouassier AM, Blanco-Bose W, Evans M, et al. Urolithin A improves muscle strength, exercise performance, and biomarkers of mitochondrial health in a randomized trial in middle-aged adults. Cell Rep Med 2022; 3(5): 100633. doi: 10.1016/j.xcrm.2022.100633 35584623 PMC9133463

[19] Zhao H, Song G, Zhu H, Qian H, Pan X, Song X, et al. Pharmacological effects of urolithin A and its role in muscle health and performance: current knowledge and prospects. Nutrients 2023; 15(20): 4441–4456. doi: 10.3390/nu15204441 37892516 PMC10609777

[20] Tao H, Li W, Zhang W, Yang C, Zhang C, Liang X, et al. Urolithin A suppresses RANKL-induced osteoclastogenesis and postmenopausal osteoporosis by, suppresses inflammation and downstream NF-kappaB activated pyroptosis pathways. Pharmacol Res 2021; 174: 105967. doi: 10.1016/j.phrs.2021.105967 34740817

[21] Tao H, Tao Y, Yang C, Li W, Zhang W, Li X, et al. Gut metabolite urolithin A inhibits osteoclastogenesis and senile osteoporosis by enhancing the autophagy capacity of bone marrow macrophages. Front Pharmacol 2022; 13: 875611. doi: 10.3389/fphar.2022.875611 35645801 PMC9135380

[22] Fu X, Gong LF, Wu YF, Lin Z, Jiang BJ, Wu L, et al. Urolithin A targets the PI3K/Akt/NF-kappaB pathways and prevents IL-1beta-induced inflammatory response in human osteoarthritis: in vitro and in vivo studies. Food Funct 2019; 10(9): 6135–46. doi: 10.1039/c9fo01332f 31497826

[23] Chen L, Yang J, Cai Z, Huang Y, Xiao P, Chen H, et al. Mitochondrial-oriented injectable hydrogel microspheres maintain homeostasis of chondrocyte metabolism to promote subcellular therapy in osteoarthritis. Research 2024; 7: 0306. doi: 10.34133/research.0306 38274127 PMC10809599

[24] Liu H, Kang H, Song C, Lei Z, Li L, Guo J, et al. Urolithin A inhibits the catabolic effect of TNFalpha on nucleus pulposus cell and alleviates intervertebral disc degeneration in vivo. Front Pharmacol 2018; 9: 1043. doi: 10.3389/fphar.2018.01043 30283339 PMC6157327

[25] Lin J, Zhuge J, Zheng X, Wu Y, Zhang Z, Xu T, et al. Urolithin A-induced mitophagy suppresses apoptosis and attenuates intervertebral disc degeneration via the AMPK signaling pathway. Free Radic Biol Med 2020; 150: 109–19. doi: 10.1016/j.freeradbiomed.2020.02.024 32105828

[26] Li Y, Zhuang Q, Tao L, Zheng K, Chen S, Yang Y, et al. Urolithin B suppressed osteoclast activation and reduced bone loss of osteoporosis via inhibiting ERK/NF-kappaB pathway. Cell Prolif 2022; 55(10): e13291. doi: 10.1111/cpr.13291 35708050 PMC9528769

[27] Qu Z, An H, Feng M, Huang W, Wang D, Zhang Z, et al. Urolithin B suppresses osteoclastogenesis via inhibiting RANKL-induced signalling pathways and attenuating ROS activities. J Cell Mol Med 2022; 26(16): 4428–39. doi: 10.1111/jcmm.17467 35781786 PMC9357644

[28] Wei W, Peng C, Gu R, Yan X, Ye J, Xu Z, et al. Urolithin A attenuates RANKL-induced osteoclastogenesis by co-regulating the p38 MAPK and Nrf2 signaling pathway. Eur J Pharmacol 2022; 921: 174865. doi: 10.1016/j.ejphar.2022.174865 35231470

[29] Bai J, Si G, Wang R, Su S, Fan J, He X, et al. Gut metabolite indoleacrylic acid suppresses osteoclast formation by AHR mediated NF-kappaB signaling pathway. Int J Biol Sci 2026; 22(2): 951–69. doi: 10.7150/ijbs.124766 41522356 PMC12781171

[30] Bai J, Han G, Fan J, Wang R, Su S, Sun A, et al. Gut microbial metabolite alleviates osteoporosis by attenuating AKT-NFATc1 signaling pathway and ROS production. Free Radic Biol Med 2026; 243: 351–66. doi: 10.1016/j.freeradbiomed.2025.11.054 41290102

[31] Bai J, Xu J, Hang K, Kuang Z, Ying L, Zhou C, et al. Glycyrrhizic acid promotes osteogenic differentiation of human bone marrow stromal cells by activating the Wnt/beta-Catenin signaling pathway. Front Pharmacol 2021; 12: 607635. doi: 10.3389/fphar.2021.607635 33935702 PMC8085383

[32] Liu S, Faitg J, Tissot C, Konstantopoulos D, Laws R, Bourdier G, et al. Urolithin A provides cardioprotection and mitochondrial quality enhancement preclinically and improves human cardiovascular health biomarkers. iScience 2025; 28(2): 111814. doi: 10.1016/j.isci.2025.111814 40034121 PMC11875685

[33] Zhao H, Zhu H, Yun H, Liu J, Song G, Teng J, et al. Assessment of Urolithin A effects on muscle endurance, strength, inflammation, oxidative stress, and protein metabolism in male athletes with resistance training: an 8-week randomized, double-blind, placebo-controlled study. J Int Soc Sports Nutr 2024; 21(1): 2419388. doi: 10.1080/15502783.2024.2419388 39487653 PMC11536656

[34] Liu T, Zhang L, Joo D, Sun S-C. NF-kappaB signaling in inflammation. Signal Transduct Target Ther 2017; 2: 17023. doi: 10.1038/sigtrans.2017.23 29158945 PMC5661633

[35] Soysa NS, Alles N. NF-kappaB functions in osteoclasts. Biochem Biophys Res Commun 2009; 378(1): 1–5. doi: 10.1016/j.bbrc.2008.10.146 18992710

[36] Iotsova V, Caamano J, Loy J, Yang Y, Lewin A, Bravo R. Osteopetrosis in mice lacking NF-kappaB1 and NF-kappaB2. Nat Med 1997; 3(11): 1285–9. doi: 10.1038/nm1197-1285 9359707

[37] Alblowi J, Kayal RA, Siqueira M, McKenzie E, Krothapalli N, McLean J, et al. High levels of tumor necrosis factor-alpha contribute to accelerated loss of cartilage in diabetic fracture healing. Am J Pathol 2009; 175(4): 1574–85. doi: 10.2353/ajpath.2009.090148 19745063 PMC2751554

[38] Moussa MR, Fan N, Birk J, Provatas AA, Mehta P, Hatano Y, et al. Systemic inflammation and the inflammatory context of the colonic microenvironment is improved by urolithin A. Cancer Prev Res (Phila) 2025;18(4): 235–250. doi: 10.1158/1940-6207.CAPR-24-0383 39995164 PMC11979956

[39] Lin YH, Chen CY, Chen KH, Kuo TY, Lin TL, Shie MY. Lithium-doped calcium silicate cement regulates the immune microenvironment and promotes M2 macrophage polarization for enhancing bone regeneration. J Biol Eng 2025; 19(1): 3. doi: 10.1186/s13036-024-00467-8 39762916 PMC11705739

