# Supplementary material for: Urolithin A supplementation alleviates osteogenic disfunction and promotes bone fracture healing in inflammatory environments
Source: Food Nutr Res. 2026 May 21;70:10.29219/fnr.v70.13033. doi: 10.29219/fnr.v70.13033 (PMC13224938; doi:10.29219/fnr.v70.13033)
Supplement: Supplementary file 1 [file FNR-70-13033-s1.docx]

Supplementary material


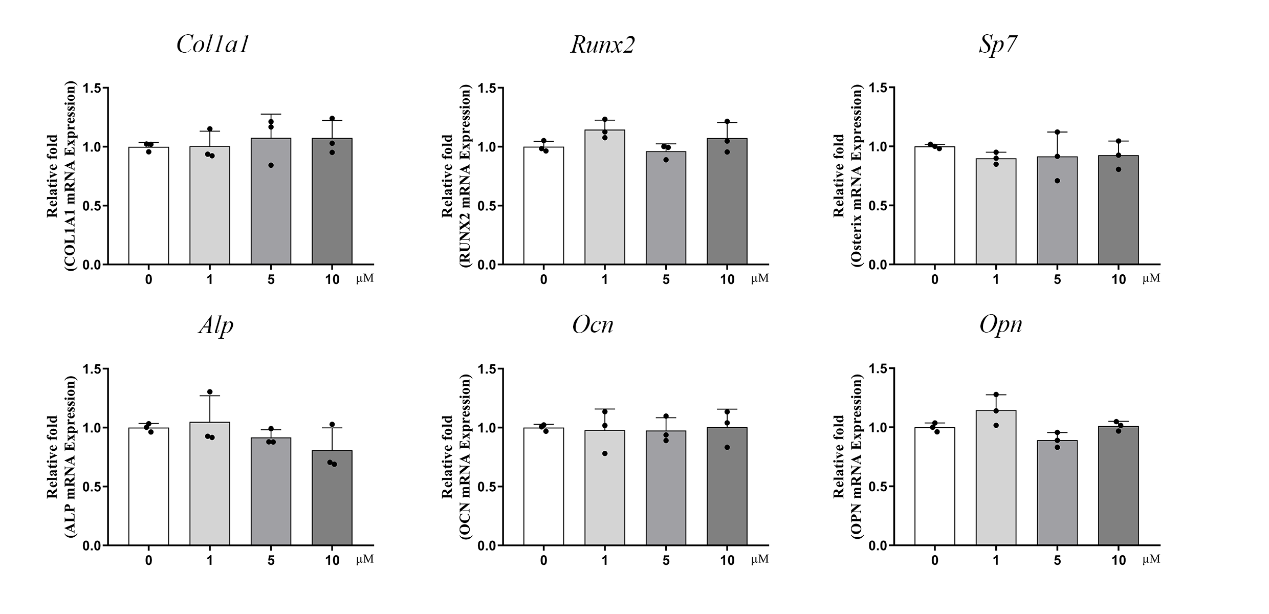


Figure S1: The osteogenic related proteins were measured by western blot after 5 days OIM. mBMSCs were induced under TNF-α with or without UA. All of the experiments were independently accomplished by three times. *p<0.05, **p<0.01 compared to the control group.
